# Powder Bed Fusion–Laser Beam of IN939: The Effect of Process Parameters on the Relative Density, Defect Formation, Surface Roughness and Microstructure

**DOI:** 10.3390/ma17133324

**Published:** 2024-07-05

**Authors:** Merve Nur Doğu, Muhannad Ahmed Obeidi, Hengfeng Gu, Chong Teng, Dermot Brabazon

**Affiliations:** 1 I-Form Advanced Manufacturing Research Centre, Dublin City University, D09 V209 Dublin, Ireland; 2 Advanced Processing Technology Research Centre, Dublin City University, D09 V209 Dublin, Ireland; 3 School of Mechanical & Manufacturing Engineering, Dublin City University, D09 V209 Dublin, Ireland; 4 Ansys Inc., 6975 Union Park Avenue, Suite 663, Cottonwood Heights, UT 84047, USA

**Keywords:** powder bed fusion–laser beam, IN939, relative density, defect formation, microstructure, surface roughness

## Abstract

This study investigates the effects of process parameters in the powder bed fusion–laser beam (PBF-LB) process on IN939 samples. The parameters examined include laser power (160, 180, and 200 W), laser scanning speed (400, 800, and 1200 mm/s), and hatch distance (50, 80, and 110 μm). The study focuses on how these parameters affect surface roughness, relative density, defect formation, and the microstructure of the samples. Surface roughness analysis revealed that the average surface roughness (Sa) values of the sample ranged from 4.6 μm to 9.5 μm, while the average height difference (Sz) varied from 78.7 μm to 176.7 μm. Furthermore, increasing the hatch distance from 50 μm to 110 μm while maintaining constant laser power and scanning speed led to a decrease in surface roughness. Relative density analysis indicated that the highest relative density was 99.35%, and the lowest was 93.56%. Additionally, the average porosity values were calculated, with the lowest being 0.06% and the highest reaching 9.18%. Although some samples had identical average porosity values, they differed in porosity/mm^2^ and average Feret size. Variations in relative density and average porosity were noted in samples with the same volumetric energy density (VED) due to different process parameters. High VED led to large, irregular pores in several samples. Microcracks, less than 50 μm in length, were present, indicating solidification cracks. The microstructural analysis of the XZ planes revealed arc-shaped melt pools, columnar elongated grains aligned with the build direction, and cellular structures with columnar dendrites. This study provides insights for optimizing PBF-LB process parameters to enhance the quality of IN939 components.

## 1. Introduction

PBF-LB is a metal additive manufacturing (AM) technique offering notable advantages over traditional manufacturing methods. These include the capacity to produce intricate metal parts with geometric complexity in a single step, enabling design freedom through near-net-shape production, and reducing material waste and tooling costs. The PBF-LB process begins by spreading a layer of metal powder onto a build plate and then selectively melting the desired areas within the powder layer using a laser beam according to a 3D computer-aided design (CAD) file. This layer-by-layer production continues until the part is fully fabricated [1,2].

In the PBF-LB process, there are over 100 processing parameters to consider [3,4]. These parameters can be broadly categorized into laser-related factors (i.e., laser power and spot size); scan-related variables (including laser scanning speed, hatch distance, scanning pattern, and rotation angle); powder-related characteristics (like powder particle morphology, size, and distribution, as well as layer thickness); and macroscopic parameters such as powder bed temperature and gas flow [4,5]. The laser power, layer thickness, laser scanning speed, and hatch distance are among the most extensively studied process parameters in the PBF-LB process. Laser power controls the energy transferred from the system to the powder, while layer thickness determines the height of each molten powder layer. Laser scanning speed dictates the rate at which the laser moves across the powder surface, and the hatch distance affects the degree of overlap between adjacent laser paths. These parameters play critical roles in determining the quality and characteristics of the fabricated parts [6,7]. 

Despite its advantages, the PBF-LB process can still result in certain unavoidable defects when improper scanning parameters and insufficient powder melting occur. These defects involve mechanical properties and impede large-scale industrial commercialization. They include partially melted powder, undesired microstructures, poor surface finish, porosity defects, balling defects, high residual stress, surface and internal cracks, and inadequate bonding between layers. Additionally, pore defects in the PBF-LB process can be categorized based on their formation mechanisms. These defects include gas pores, which can be categorized into keyhole pores and powder feedstock pores, as well as lack of fusion (LOF) defects, such as intertrack LOF, interlayer LOF, and LOF caused by spattering. Unstable melt pools have been shown as the primary reason for these defects [2,5,7,8]. Moreover, rapid solidification, high cooling rates (10^5^–10^7^ K/s), and repeated thermal cycles lead to non-equilibrium solidification, causing residual stress [9,10].

IN939 is a precipitation-hardenable Ni-base superalloy, primarily strengthened by the formation of the L12-ordered γ′ phase (Ni_3_(Al, Ti)). Originally, it was developed in the late 1960s as a cast alloy to meet the demand for a robust, highly corrosion-resistant material capable of prolonged operation at temperatures reaching 850 °C. It has found extensive application in higher-temperature applications within aerospace engines, particularly in hot sections such as turbine blades and nozzle guide vanes, owing to its exceptional properties. The microstructure of as-cast IN939 consists of a gamma (γ) phase solid-solution matrix, with the gamma prime (γ′) phase serving as the primary strengthening component. 

While IN939 was initially developed as a cast alloy [11,12,13], recent attention has been given to its production using the PBF-LB process [1,14,15,16,17,18,19,20,21,22,23]. However, there are limited studies in the literature focusing on optimizing the process parameters for achieving the desired relative density in IN939 produced via the PBF-LB process. Table 1 summarizes the details of existing studies on process parameter optimization for IN939 fabricated using PBF-LB, revealing variations in powder suppliers, PBF-LB machines, and process parameters used. It is important to note that powder characteristics, such as particle size distribution, flowability, chemistry, and morphology, significantly influence the build quality and porosity distribution in the PBF-LB process. Additionally, PBF-LB machines themselves can introduce defects due to issues with the laser beam scanning system, build chamber environment, powder spreading system, and baseplate [5,24,25]. For instance, Obeidi et al. [26] reported significant variations in the mechanical performance and properties of 316L samples produced on different PBF-LB machines despite using the same process parameters.

Although IN939 is a well-established Ni-base superalloy, its application in the PBF-LB process is relatively new, necessitating further research to understand the effects of this process on IN939. This study aims to address this gap by systematically investigating the influence of key process parameters, such as laser power, laser scanning speed, and hatch distance, on the relative density, defect formation, surface roughness, and microstructure of IN939 fabricated by the PBF-LB process. By optimizing these parameters, this research seeks to enhance the performance and reliability of IN939 components produced using PBF-LB technology, contributing to the advancement of additive manufacturing techniques for high-performance superalloys. 

## 2. Materials and Methodology

### 2.1. IN939 Fabrication Using the PBF-LB Process

We employed gas-atomized IN939 powder whose particle size distribution is between 17.4 μm and 52 μm (Truform 939-N65, Praxair Surface Technologies, Speedway, IN, USA) to produce the IN939 samples. The chemical composition of the powder is given in Table 2, and a detailed powder characterization is presented in a prior study conducted by the authors [1].

An Aconity MINI (GmbH) metal 3D printer equipped with a Ytterbium fiber laser from IPG (Herzogenrath, Germany), model YLR-200-WC-Y11, 2011 series, with a wavelength of 1068 nm, was used to fabricate IN939 samples. All fabrication procedures were carried out under a protective argon atmosphere maintained using 99.999% pure argon gas, ensuring that oxygen levels remained below 20 ppm. Additionally, CK45 steel was used as a build plate for all prints.

**Figure 1:**
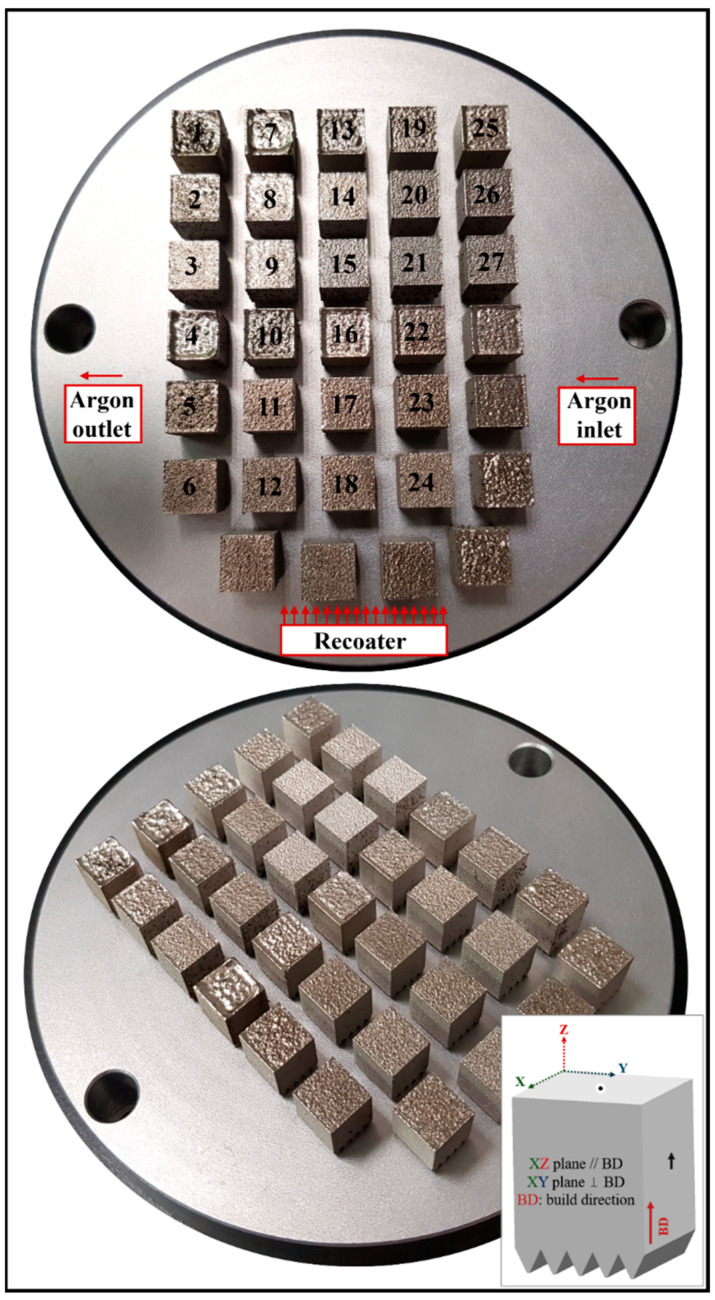
Images of the build plate after fabrication and a schematic of the as-built IN939 samples. The XZ plane (parallel to the build direction) and the XY plane (perpendicular to the build direction) are indicated with arrows and dots, respectively.

IN939 cubic samples (10 mm × 10 mm × 10 mm with 2 mm support) were fabricated with the Aconity MINI printer. Figure 1 presents images of the build plate post-fabrication, along with a schematic representation of the as-built samples. A full factorial design of experiment (DoE) model with 3 factors at 3 levels (3^3^) was created to analyze the impact of input processing parameters on output characteristics. The input parameters included laser power, laser scanning speed, and hatch distance, and the PBF-LB process parameters used in this study are given in Table 3. The full factorial DoE combination of the PBF-LB process parameters and VED values are listed in Table 4 with the corresponding sample number. The input VED [27] was calculated according to Equation (1) as follows:

(1) $$\mathrm{VED}=\frac{P}{V\;\times \;h\;\times \;t}\;(J/{\mathrm{mm}}^{3})$$

where P represents laser power (W), V stands for laser scanning speed (mm/s), h presents hatch distance (μm), and t denotes layer thickness (μm).

### 2.2. Surface Roughness Measurement

Surface roughness measurements of the as-built samples were conducted using Bruker ContourGT (Billerica, MA, USA), focusing on the XZ planes over a 2 mm × 2 mm area. The Sa value, indicating the arithmetical mean height, was utilized to quantify surface roughness. Additionally, the Sz value represents the summation of the maximum peak height and maximum pit depth. A rainbow scale bar, ranging from +100 μm to −87 μm, was employed to enhance the visualization of surface roughness disparities among the samples.

### 2.3. Relative Density and Porosity Measurements

The relative density of the as-built samples was measured with Archimedes’ method using a Sartorius Entris II Essential BCE124I-1S analytical balance with an accuracy and repeatability of ±0.1 mg according to ASTM B311-17 [28]. The measurements were repeated three times for each sample to obtain the average relative density value of each sample. Before the measurements, the as-built samples were ground lightly to flat on all faces with 80 SiC abrasive paper and cleaned thoroughly. Ethanol (Lenox, Dublin, Ireland, 99.99%) was used as the fluid. Additionally, the theoretical density of a fully dense IN939 was taken as 8.15 g/cm^3^ to calculate the relative density values of the samples [29]. 

Conventional optical microscopy was employed to analyze the distribution of porosity. Optical images of the as-polished cross-sections, captured from both XZ and XY planes, were taken utilizing the stitching capability of the Keyence 3D optical microscope. For porosity calculation, the stitched optical images, including at least 20 images for XZ planes and 10 images for XY planes, were analyzed using ImageJ 1.54i software.

The response surface method (RSM) is a collection of mathematical and statistical techniques used for modeling and predicting the output response. To analyze the relationship between input laser process parameters and relative density (%) and surface roughness (μm), the RSM using an experimental design was employed by using Design-Expert 13 software. 

### 2.4. Microstructural Characterization

The as-built samples were precision-cut to investigate both XZ and XY planes (the XZ plane is parallel to the building direction, and the XY plane is perpendicular to the building direction) and were hot-mounted with Bakelite. Then, the mounted samples were automatically ground using conventional SiC grinding papers (up to 1200 grit sizes) and polished with progressively finer diamond suspensions (9, 3, and 1 μm) using a Struers Tegramin-20 machine (Struers, Catcliffe, UK). After that, the as-polished samples were etched with the Glyceregia reagent (15 mL HCl, 10 mL glycerol, and 5 mL HNO_3_) for further microstructural examination. For microstructural examinations, a Keyence VHX2000E optical 3D digital microscope (OM) (Osaka, Japan) and Zeiss EVO LS-15 Scanning Electron Microscope (SEM) (Oberkochen, Germany) were utilized. Zeiss EVO LS 15, equipped with an Oxford EDS detector, was used for energy dispersive X-ray spectroscopy (EDS) analysis (an acceleration voltage of 15 kV, 1.0 nA probe current, WD: 8.5 mm).

## 3. Results

### 3.1. Surface Roughness

**Figure 2:**
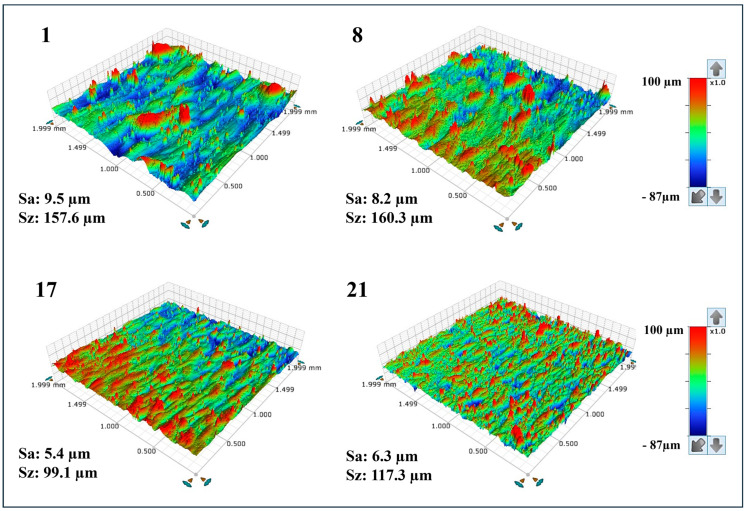
Surface roughness profiles of the selected as-built IN939 samples (samples 1, 8, 17, and 21).

**Figure 3:**
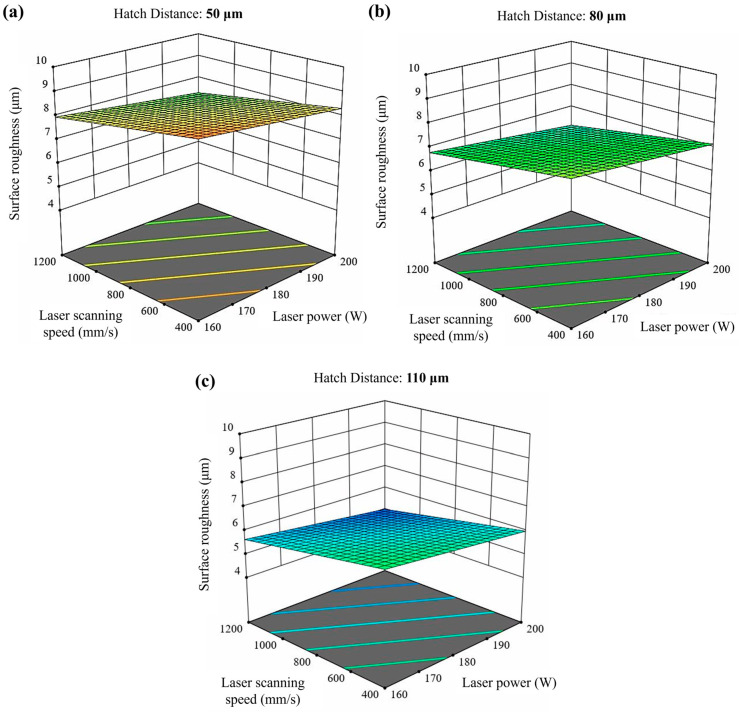
RSM graphs of the surface roughness (μm) versus the different input processing parameters. Hatch distance: (**a**) 50 μm, (**b**) 80 μm and (**c**) 110 μm.

**Figure A1:**
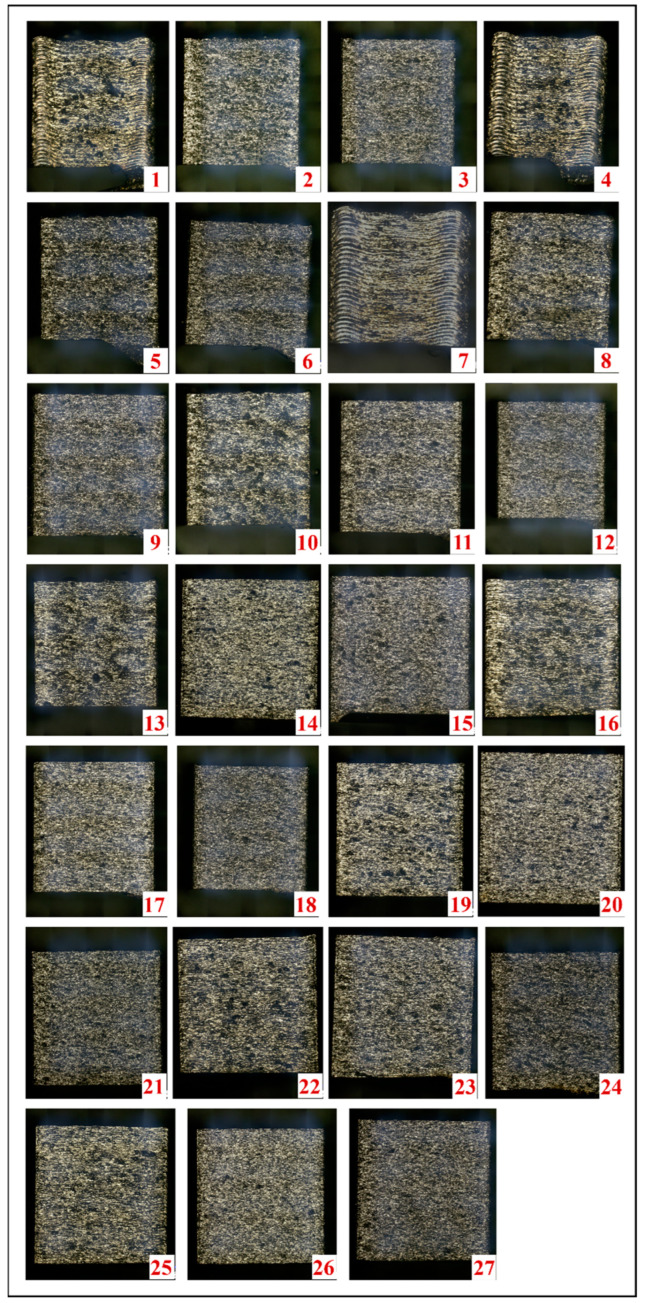
Optical images of the XZ planes of the as-built IN939 samples (1–27).

Table 5 displays the average surface roughness (Sa), ranging from 4.6 μm to 9.5 μm, and the maximum height (Sz), ranging from 78.7 μm to 176.7 μm, for the XZ planes of the as-built samples. Among the samples, the lowest Sa value (4.6 μm) belongs to sample 26, whereas samples 1 and 2 have the highest Sa value (9.5 μm). Moreover, Figure 2 shows the surface roughness profiles of the selected as-built IN939 samples (samples 1, 8, 17, and 21). Additionally, optical images of the XZ planes of the as-built IN939 samples are presented in Figure A1 in Appendix A. It can be seen from the optical images that PBF-LB process parameters have a direct influence on the dimensional accuracy and surface roughness. High VED resulted in the formation of devil horns due to over-melting. This defect was particularly pronounced in samples 1, 4, and 7, which had the highest VED values. Moreover, a strong correlation was found between the input processing parameters and the surface roughness (μm), as shown in Figure 3. Each graph displays the full range of two process parameters versus the surface roughness (μm), with the other parameters held constant. The model developed for the effect of the laser input power, laser scanning speed, and hatch distance on the resulting surface roughness was statistically significant (*p*-value < 0.0008).

### 3.2. Relative Density 

**Figure 4:**
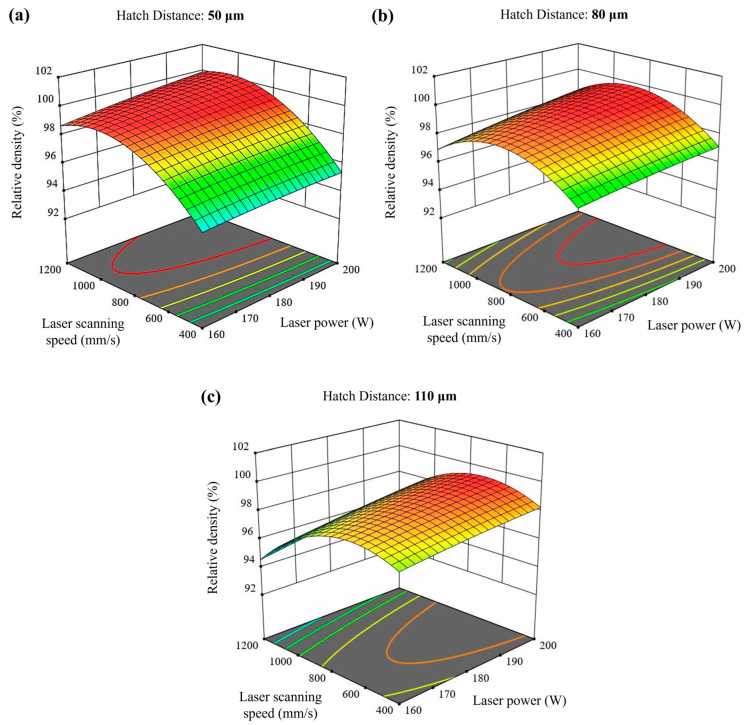
RSM graphs of the relative density (%) versus the different input processing parameters. Hatch distance: (**a**) 50 μm, (**b**) 80 μm and (**c**) 110 μm.

**Figure A2:**
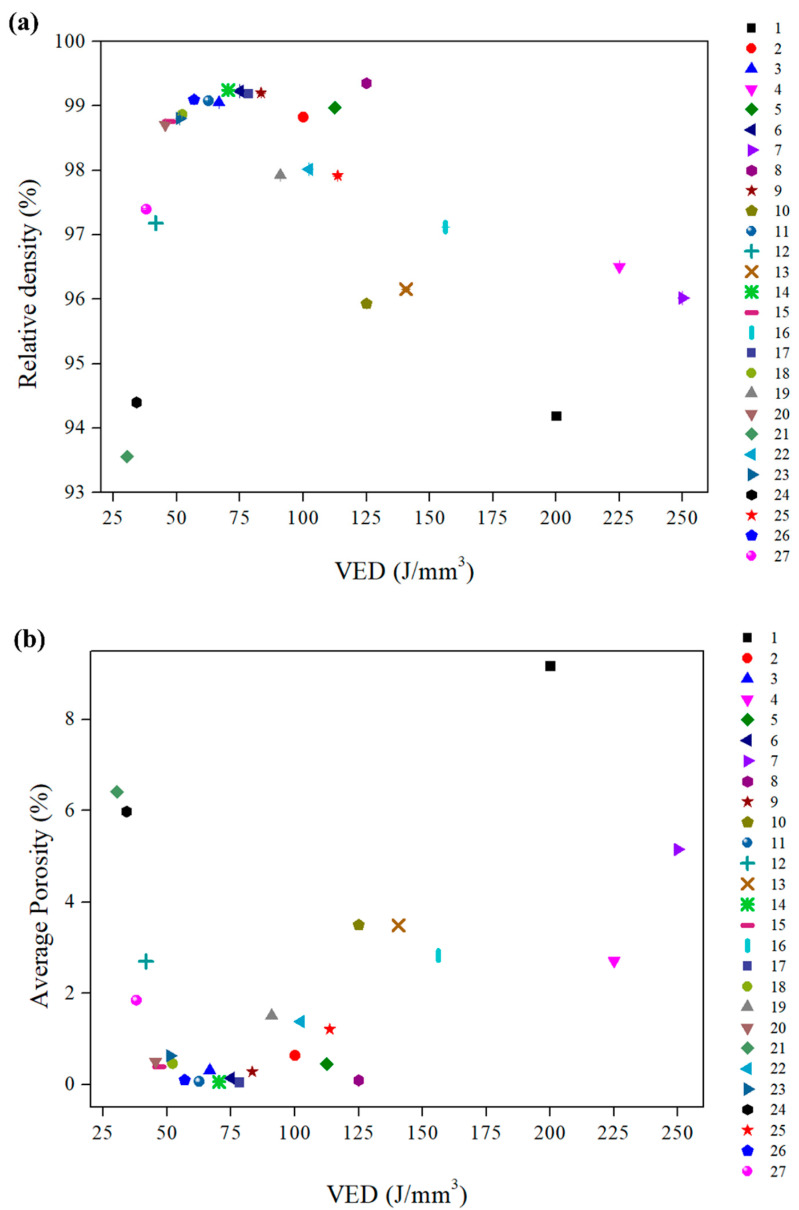
(**a**) Relative density (%) and (**b**) average porosity (%) versus VED (J/mm^3^) graphs of the as-built samples.

Relative density values are depicted in Table 6. Additionally, Figure A2a in Appendix A shows the relative density (%) versus VED (J/mm^3^) graph. The results indicated that sample 8 exhibited the highest relative density at 99.35%, whereas sample 21 showed the lowest relative density at 93.56%. Furthermore, samples 14, 6, 9, and 17 also exhibited high relative densities at 99.25%, 99.23%, 99.20%, and 99.20%, respectively. On the other hand, sample 1 had a very low relative density (94.19%) after sample 21. Moreover, the same VED (125 J/mm^3^) values, obtained using different process parameters, resulted in different relative densities for samples 8 and 10. Furthermore, a strong correlation was found between the input processing parameters and the relative density (%), as shown in Figure 4. Each graph displays the full range of two process parameters versus the relative density (%), with the other parameters held constant. The model developed for the effect of the laser input power, laser scanning speed, and hatch distance on the resulting relative density was statistically significant (*p*-value < 0.0001).

### 3.3. Defect Formation

**Figure 5:**
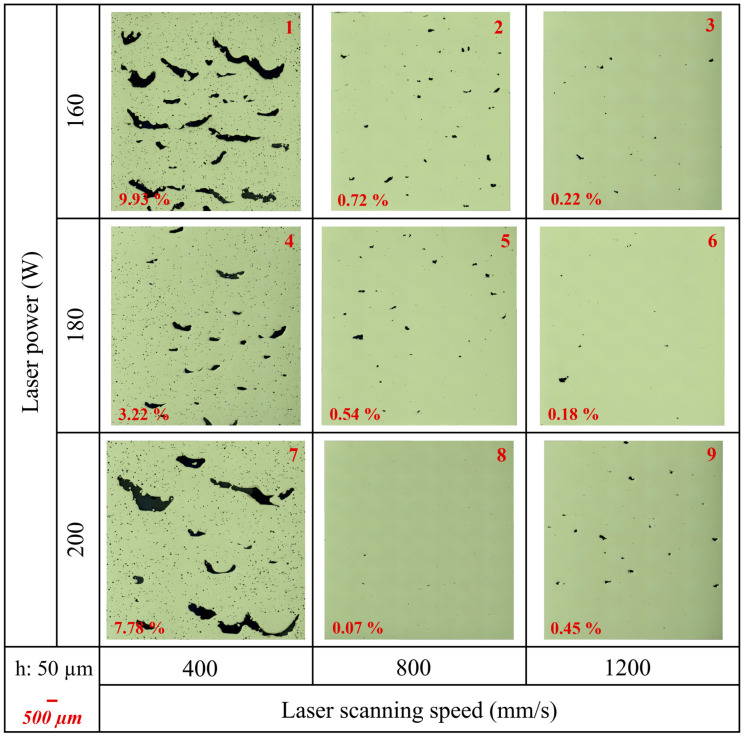
The as-polished optical micrographs of as-built samples (1–9) in the XZ plane (parallel to the build direction). Porosity (%) values are indicated on the micrographs (hatch distance: 50 μm).

**Figure 6:**
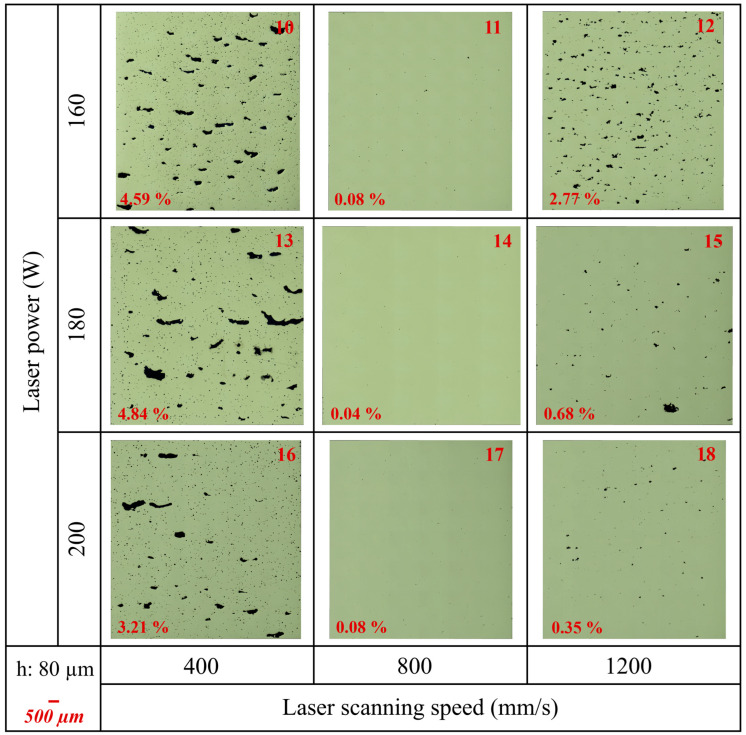
The as-polished optical micrographs of as-built samples (10–18) in the XZ plane (parallel to the build direction). Porosity (%) values are indicated on the micrographs (hatch distance: 80 μm).

**Figure 7:**
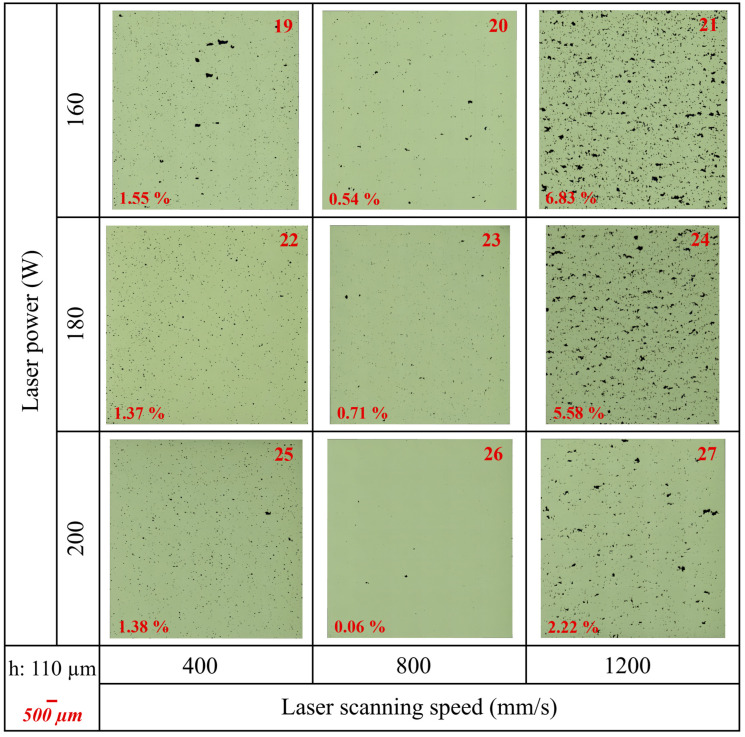
The as-polished optical micrographs of as-built samples (19–27) in the XZ plane (parallel to the build direction). Porosity (%) values are indicated on the micrographs (hatch distance: 110 μm).

**Figure A3:**
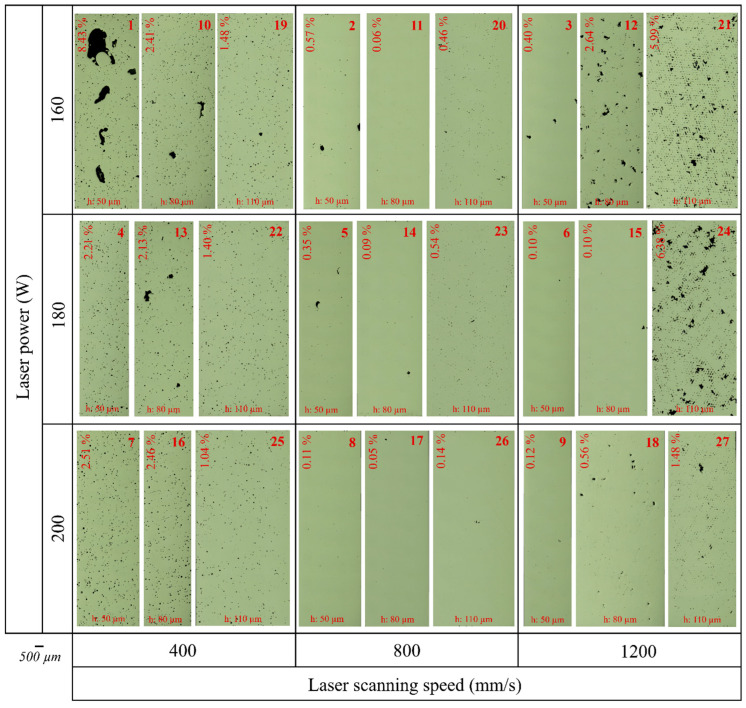
The as-polished optical micrographs of as-built samples (1–27) in the XY plane (perpendicular to the build direction). Porosity (%) values are indicated on the micrographs.

The porosity percentages of the samples were calculated by including all void defects, such as gas porosity and lack of fusion (LOF) defects using ImageJ. Table 7 presents the results of the ImageJ porosity analysis for the samples, along with the average results, including average porosity (%), pores/mm^2^, and average Feret size (μm). Additionally, Figure A2b in Appendix A shows the average porosity (%) versus the VED (J/mm^3^) graph. Furthermore, Figure 5, Figure 6, and Figure 7 show the as-polished optical micrographs of the XZ planes of the samples. In addition to this, the results of the ImageJ porosity analysis for the XZ and XY planes of the samples are given in Table A1 in Appendix A, and the as-polished optical micrographs of the XY planes of the samples are displayed in Figure A3 in Appendix A. 

A higher pores/mm^2^ value indicates a higher concentration of pores, which can decrease mechanical properties such as strength and fatigue resistance. Conversely, a lower pores/mm^2^ value suggests fewer pores, indicating better material quality and integrity. Additionally, the average Feret size provides insight into pore dimensions, with a larger average Feret size indicating the presence of larger pores and a smaller average Feret size indicating predominantly smaller pores. According to the results, sample 1 exhibited the highest average porosity (%) at 9.18%, while samples 14 and 17 showed the lowest average porosity at 0.06%. Additionally, the average porosity (%) for sample 11 was 0.07%, closely resembling that of samples 14 and 17. Although samples 14 and 17 had the same average porosity (%) values, their porosity/mm^2^ and average Feret size values were different (Table 7). On the other hand, samples 8 and 10 had completely different average porosity (%) values at 0.09% and 3.50% even though they had the same VED value. Very high VED resulted in large, irregular pores for samples 1, 4, and 7 (Figure 5) and samples 10, 13, and 16 (Figure 6). Additionally, these samples contained a high number of small pores, leading to high pores/mm^2^ values. However, the combination of very large and very small pores reduced the average Feret size. On the other hand, the lowest VED, which was 30.3 J/mm^3^, belonged to sample 21. This sample exhibited the lowest relative density and high porosity due to insufficient energy for melting (Figure 7). Additionally, samples 24 and 27 exhibited low relative density and high porosity due to their low VED values.

### 3.4. Microstructure

**Figure 8:**
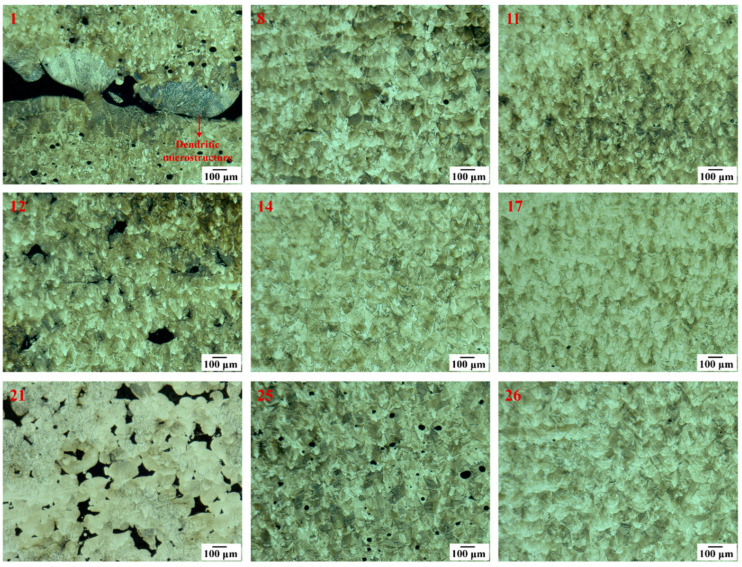
Optical micrographs of the XZ planes of the selected as-built samples (1, 8, 11, 12, 14, 17, 21, 25, and 26).

**Figure A4:**
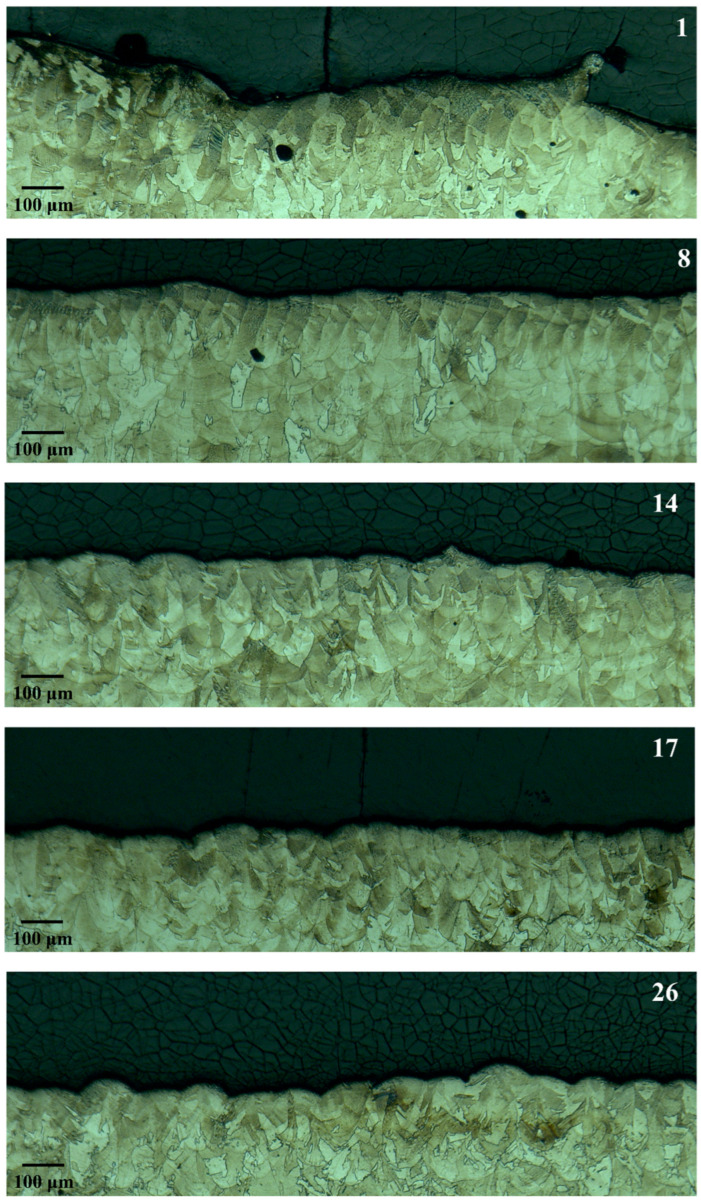
Optical micrographs of the XZ planes of the selected as-built samples (1, 8, 14, 17, and 26).

The grain structures of the selected samples (1, 8, 11, 12, 14, 17, 21, 25, and 26) can be seen in the optical micrographs (Figure 8). The Gaussian energy distribution of the laser beam in the PBF-LB process results in arc-shaped melt pools in the XZ planes, which are parallel to the build direction. The arc-shaped melt pools, along with a microstructure composed of elongated columnar grains along the build direction, were observed in the XZ planes of the samples. Furthermore, samples fabricated with high VED (i.e., sample 1) had regions with a dendritic microstructure. The cross-section micrographs of the top layers of selected samples (1, 8, 14, 17, and 26) are displayed in Figure A4 in Appendix A. The micrographs reveal that melt pools with a higher depth/width aspect ratio (keyhole melting mode) were formed in samples 1 and 8 due to the higher applied VED, indicating that a dynamic melt pool motion occurred during melting.

**Figure 9:**
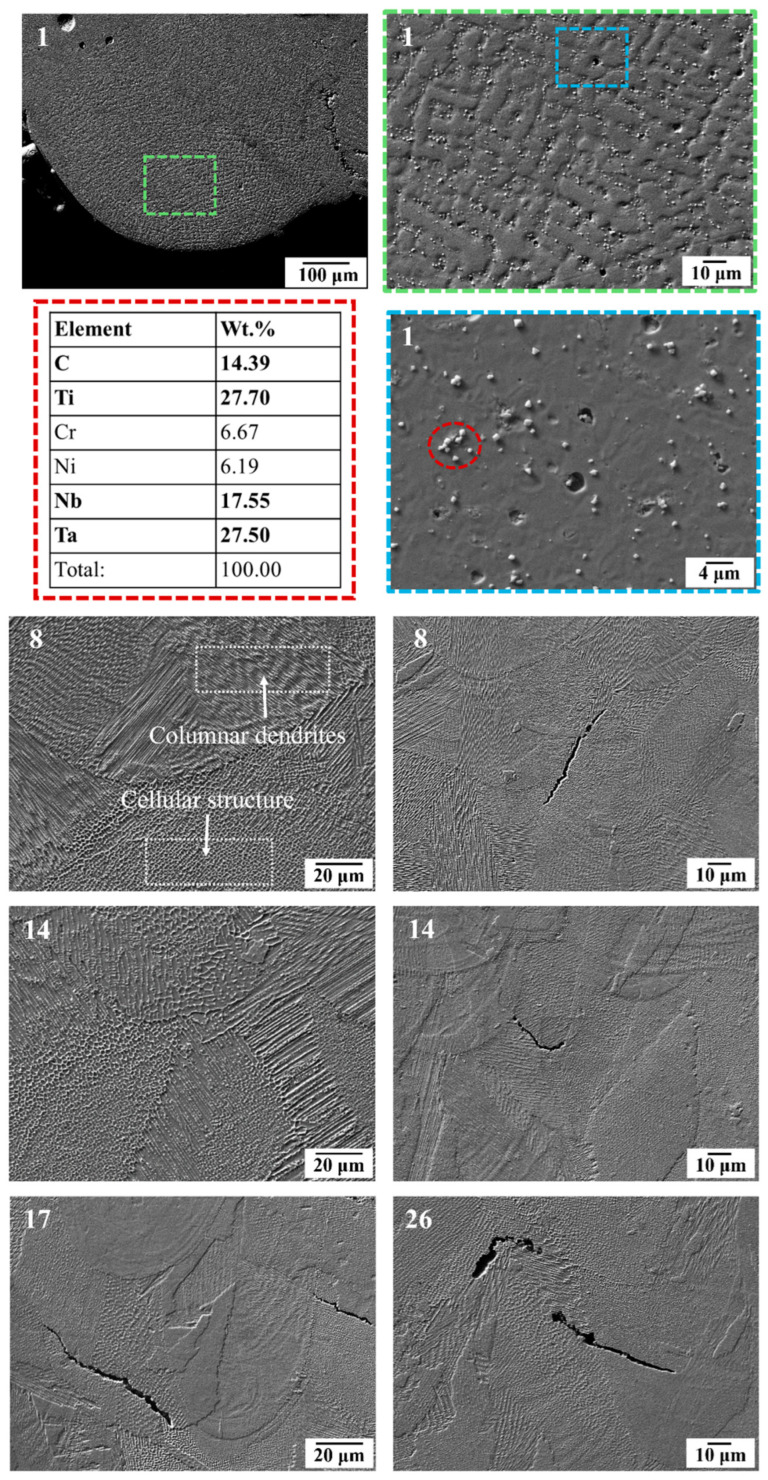
SEM images of the XZ planes of the selected as-built samples (1, 8, 14, 17, and 26), along with EDS results of the MC-type carbides.

SEM images of the selected samples (1, 8, 14, 17, and 26) show cellular structures and columnar dendrites within the microstructure (Figure 9). Furthermore, during EDS analysis of the dendritic region of sample 1, fine irregular-shaped MC-type (rich in Ti, Ta, and Nb) carbides were observed. During SEM analysis, a few microcracks (less than 50 microns in length) were observed in the samples, although these microcracks were not visible in as-polished micrographs. The authors recommend conducting the SEM examination of samples after etching to ensure a thorough assessment of the cracks. It should be noted that some microcracks, invisible after polishing, became apparent after etching. Therefore, this additional step ensures a more comprehensive assessment of crack formation.

## 4. Discussion

Among the PBF-LB process parameters, laser power, laser scanning speed, hatch distance, layer thickness, and scanning strategy are the main factors [30]. The combination of these process parameters significantly influences melt pool geometry, local microstructure, defect size, and defect morphology [2]. The current study demonstrates that the relative density, defect formation, surface roughness, and microstructure of as-built IN939 samples can be directly controlled by adjusting the PBF-LB process parameters. In the literature, an effective process window, defined by LOF, keyhole, and bead-up porosity boundaries, has been established to optimize process parameters for the PBF-LB process, enabling the production of parts with nominally full density. It should be noted that even if a sample is fully dense (volumetric density > 99.9%), it may still have large defects [2,5]. In our study, sample 6 is a good example of this. Although it had a high relative density (99.23%) and low average porosity (0.14%), the average Feret size was calculated as 108.3 μm. This can also be seen from the as-polished micrographs (Figure 5 and Figure A3 in Appendix A).

The combination of high laser power, low laser scanning speed, and small layer thickness results in excessive energy, leading to a highly fluctuated molten pool. This melt pool exhibits a keyhole melting mode, often resulting in keyhole porosity [7]. Keyhole formation occurs in four stages: liquid vaporization in the melt pool, the depression of the liquid surface, instability, and keyhole formation. The formation of a keyhole indicates that the melt pool enters a volatile state, where surface tension, drag force, recoil pressure, and other forces are coupled in the molten pool. This dynamic environment causes continuous keyhole fluctuations, which play a vital role in the formation of keyhole pores. Additionally, keyhole melt pools have often a “J” shape [8]. Aboulkhair et al. [31] reported that keyhole pores are irregularly shaped and larger than 100 µm. Specifically, in this study, samples 1, 4, 7, 10, 13, and 16 exhibited very large, irregular keyhole pores attributed to high VED, along with small spherical pores. On the other hand, low laser power combined with large layer thickness and high laser scanning speed can generate insufficient energy. This often results in high surface tension, unmelted powder, and poor wetting of the molten pool, leading to balling and dimensional errors. Intertrack LOF, interlayer LOF, and spattering-induced LOF are the three main types of LOF defects. Intertrack LOF stems from an inadequate melt pool overlap due to factors like melt pool shape, size, and hatch spacing. Interlayer LOF arises from incomplete bonding between layers, primarily due to low laser energy density, limiting melt pool depth and flow. Spattering-induced LOF occurs when spatters deposited on the part’s surface hinder uniform powder spreading, leading to numerous LOF defects [7,8]. In particular, samples 12, 21, 24, and 27 are good examples of LOF defects due to insufficient energy. Furthermore, hatch distance significantly influences the overlapping rate of scan tracks, impacting densification and surface roughness. A high hatch space can cause insufficient overlap between adjacent tracks, leaving unmelted powder on the layer. Conversely, a low hatch space can result in the excessive melting of the previous track, leading to a rough surface and a heat-affected zone [7]. RSM graphs for surface roughness (Figure 3) and relative density (Figure 4) clearly show the importance of the hatch distance. 

It should be noted that relative densities for PBF-LB materials produced using different processing parameters can vary by up to 5%, despite having the same energy density. For instance, if hatch spacing is increased and layer thickness is decreased by the same proportion, the energy density remains constant, yet porosity outcomes differ [2]. Samples 8 and 10 illustrate this phenomenon. Despite having the same VED of 125 J/mm^3^, their relative densities and average porosity values differed significantly. Sample 8 exhibited a relative density of 99.35% and an average porosity of 0.09%, while sample 10 had a relative density of 95.93% and an average porosity of 3.50%. This demonstrates that VED alone does not reliably predict porosity or density outcomes in PBF-LB materials.

Spatter formation is inherently due to the nature of the PBF-LB process. Alleviating defects caused by spatter powder is challenging because the landing position of the spatter during the PBF-LB process is unpredictable. For this reason, spattering affects the microstructure, part quality, and properties of the PBF-LB materials [1]. A dendritic microstructure was observed near keyhole pores, as shown in Figure 8 (sample 1), which can be attributed to the thermal conductivity difference between the air trapped in the keyhole pores and the solid material [31]. Additionally, these dendritic regions can be partially melted spatter powder. Rapid solidification during the PBF-LB process causes the segregation of certain elements, leading to the formation of MC-type (i.e., Ti-, Ta-, and Nb-rich) and M_23_C_6_-type carbides (i.e., Cr- and W-rich M_23_C_6_ carbides). The effects of MC-type carbides on the mechanical properties of Ni-based superalloys can be either beneficial or detrimental, primarily depending on their distribution and morphology. They can negatively affect the mechanical properties of Ni-base superalloys when they act as nucleation sites for crack formation. However, when located within grains, they can act as barriers to dislocation movement, like precipitates, thereby potentially enhancing the mechanical properties [14,32,33].

Furthermore, in the PBF-LB process, rapid cooling and non-equilibrium solidification significantly affect the solidification microstructure. This microstructure is influenced by parameters such as solidification rate (R), undercooling (ΔT), and temperature gradient (G), alongside PBF-LB process parameters. The size and morphology of the solidification microstructure, whether planar, cellular, equiaxed dendritic, or columnar dendritic, are determined by G*R and G/R. Lower cooling rates result in coarser structures (G*R), while higher cooling rates lead to finer structures [34,35]. An extremely high G/R ratio results in a planar solidification morphology, while a moderate G/R ratio leads to cellular structures, and a low G/R ratio produces columnar or equiaxed dendritic structures. The PBF-LB process has high cooling rates, which can change according to process parameters used in the process, typically yielding high G/R values, which favor the formation of cellular structures [36]. Moreover, columnar dendritic structures can also be seen in the PBF-LB process [14]. The cellular structures exhibit a honeycomb-like morphology, varying with the observation direction: They appear as parallel boundaries along the building direction and as circular features on the transverse section. Consequently, the cellular structures can manifest as circles, ellipses, or parallel lines in different cross-sections, a characteristic widely observed in additively manufactured metals and alloys [36].

Superalloys like IN939, rich in Al and Ti, form the L12-ordered γ’ phase (Ni_3_(Al, Ti)) but are prone to cracking in the PBF-LB process [37]. Two primary crack types are observed in IN939 during the PBF-LB process: solidification cracks and solid-state cracks. Solidification cracks, or “hot tears”, occur in the semisolid state within the mushy zone due to interdendritic stress concentration. Solid-state cracks include strain-age cracks, ductility-dip cracks (DDCs), and cold cracks. Additionally, oxides can contribute to crack formation by causing stress concentrations, increased boundary brittleness, and constitutional liquation at the oxide–matrix interface [9,38,39,40]. In this study, a few cracks, which became visible after the etching process, were observed, and they appeared to be solidification cracks.

## 5. Conclusions

Optimizing process parameters is crucial for achieving desired properties such as defect-free samples in the PBF-LB process. This study aimed to fill the existing research gap by investigating the effects of process parameters such as laser power, laser scanning speed, and hatch distance on the relative density, defect formation, surface roughness, and microstructure of IN939 fabricated via the PBF-LB process. The main findings from the observed results are summarized as follows:

(1) The average surface roughness (Sa) of the XZ planes of the as-built samples ranged from 4.6 μm to 9.5 μm, while the average height difference (Sz) ranged from 78.7 μm to 176.7 μm. Sample 26 had the lowest Sa (4.6 μm), while samples 1 and 2 had the highest (9.5 μm).
(2) Sample 8 had the highest relative density at 99.35%, and sample 21 had the lowest at 93.56%. Samples 14, 6, 9, and 17 also showed high relative densities of 99.25%, 99.23%, 99.20%, and 99.20%, respectively. Sample 1 had a notably low relative density of 94.19%, just above sample 21. Samples 8 and 10, despite having the same VED (125 J/mm^3^), exhibited different relative densities (%) and porosity (%) due to varying process parameters.
(3) Sample 1 had the highest average porosity at 9.18%, while samples 14 and 17 had the lowest at 0.06%. Sample 11 showed a similar low porosity at 0.07%. Despite the same average porosity, samples 14 and 17 differed in porosity/mm^2^ and average Feret size. High VED resulted in large, irregular pores for samples 1, 4, 7, 10, 13, and 16. Sample 21, with the lowest VED (30.3 J/mm^3^), showed high porosity due to insufficient melting energy.
(4) The samples contained few microcracks, each less than 50 microns in length, indicative of solidification cracks.
(5) Observations in the XZ planes of the samples revealed arc-shaped melt pools and a microstructure characterized by elongated columnar grains aligned along the build direction. Additionally, cellular structures and columnar dendrites were observed within the microstructure of the samples.

## Figures and Tables

**Table 1:** Studies for process parameters optimization of IN939 fabricated by the PBF-LB.

Reference	Marchese et al. [22]	Dursun et al. [20]	Rodríguez-Barber et al. [16]
**Powder**	Gas-atomized IN939 powder (LPW Carpenter Additive)	-	Gas-atomized IN939 powder (Eckart TLS GmbH)
**PBF-LB machine**	CONCEPT Mlab Cusing R system	EOS M290	Renishaw AM400
**Laser power (W)**	95 (fixed)	200–350	250–300
**Laser speed (mm/s)**	100–2000	800–1400	1000–1750
**Hatch distance (mm)**	0.02–0.15	0.1 (fixed)	0.05–0.09
**Layer thickness (μm)**	20 (fixed)	40 (fixed)	60 (fixed)
**Scanning strategy**	Stripes of 5 mm with a rotation of 67°	-	Bidirectional scanning strategy, with a 67° rotation
**Laser mode**	Continuous (CW)	Continuous (CW)	Pulsed (PW)
**VED (J/mm^3^)**	30–320	35–109	26–100
**Preheating (°C)**	-	80	-
**Characterization**	Cubic samples	Single-tracks and cubic samples	Cubic samples

**Table 2:** The nominal chemical composition (wt.%) of the gas atomized IN939 powder.

Elements	Al	Co	Cr	Nb	Ta	Ti	W
**wt.%**	1.9	18.9	22.8	1.0	1.4	3.8	2.0
	**Zr**	**Ni**	**B**	**C**	**O**	**N**	
	0.028	Bal.	0.004	0.16	0.014	0.009	

**Table 3:** PBF-LB process parameters used in the present study and their levels.

Process Parameters	Level-1	Level-2	Level-3
Laser power (W)	160	180	200
Laser scanning speed (mm/s)	400	800	1200
Hatch distance (μm)	50	80	110
Layer thickness (μm)	40 (fixed)		
Spot size (μm)	80 (fixed)		
Contour (μm)	50		
Scanning strategy	Alternating bidirectional scan with 67° rotation		

**Table 4:** PBF-LB process parameters for the entire DoE, along with the sample numbers.

Sample	Laser Power (W)	Hatch Distance (μm)	Laser Scanning Speed (mm/s)	VED (J/mm^3^)
1	160	50	400	200.0
2	160	50	800	100.0
3	160	50	1200	66.7
4	180	50	400	225.0
5	180	50	800	112.5
6	180	50	1200	75.0
7	200	50	400	250.0
8	200	50	800	125.0
9	200	50	1200	83.3
10	160	80	400	125.0
11	160	80	800	62.5
12	160	80	1200	41.7
13	180	80	400	140.6
14	180	80	800	70.3
15	180	80	1200	46.9
16	200	80	400	156.3
17	200	80	800	78.1
18	200	80	1200	52.1
19	160	110	400	90.9
20	160	110	800	45.5
21	160	110	1200	30.3
22	180	110	400	102.3
23	180	110	800	51.1
24	180	110	1200	34.1
25	200	110	400	113.6
26	200	110	800	56.8
27	200	110	1200	37.9

**Table 5:** Average surface roughness (Sa) and the maximum height (Sz) values for the XZ planes of the as-built IN939 samples.

Sample	Sa (µm)	Sz (µm)	Sample	Sa (µm)	Sz (µm)
**1**	9.5	157.6	**15**	5.3	118.2
**2**	9.5	164.7	**16**	8.9	141.1
**3**	7.6	137.8	**17**	5.4	99.1
**4**	9.2	153.5	**18**	6.8	129.8
**5**	8.8	154.2	**19**	6.0	168.3
**6**	7.1	138.4	**20**	5.1	124.1
**7**	6.0	78.7	**21**	6.3	117.3
**8**	8.2	160.3	**22**	6.2	85.0
**9**	7.7	173.0	**23**	5.7	133.4
**10**	7.9	130.2	**24**	6.7	134.1
**11**	6.0	138.6	**25**	6.6	107.0
**12**	7.6	154.5	**26**	4.6	95.0
**13**	9.3	176.7	**27**	5.5	150.4
**14**	5.0	87.0			

**Table 6:** Relative density (%) values of the as-built IN939 samples (errors show 95% CI).

Sample	P (W)	h (μm)	V (mm/s)	VED (J/mm^3^)	Relative Density (%)
1	160	50	400	200.0	94.19 ± 0.009
2	160	50	800	100.0	98.83 ± 0.008
3	160	50	1200	66.7	99.05 ± 0.010
4	180	50	400	225.0	96.50 ± 0.006
5	180	50	800	112.5	98.97 ± 0.003
6	180	50	1200	75.0	99.23 ± 0.003
7	200	50	400	250.0	96.02 ± 0.007
8	200	50	800	125.0	99.35 ± 0.011
9	200	50	1200	83.3	99.20 ± 0.006
10	160	80	400	125.0	95.93 ± 0.002
11	160	80	800	62.5	99.08 ± 0.004
12	160	80	1200	41.7	97.18 ± 0.001
13	180	80	400	140.6	96.16 ± 0.007
14	180	80	800	70.3	99.25 ± 0.010
15	180	80	1200	46.9	98.76 ± 0.001
16	200	80	400	156.3	97.12 ± 0.004
17	200	80	800	78.1	99.20 ± 0.002
18	200	80	1200	52.1	98.87 ± 0.007
19	160	110	400	90.9	97.92 ± 0.003
20	160	110	800	45.5	98.71 ± 0.005
21	160	110	1200	30.3	93.56 ± 0.009
22	180	110	400	102.3	98.02 ± 0.003
23	180	110	800	51.1	98.81 ± 0.002
24	180	110	1200	34.1	94.40 ± 0.011
25	200	110	400	113.6	97.92 ± 0.013
26	200	110	800	56.8	99.10 ± 0.016
27	200	110	1200	37.9	97.40 ± 0.008

**Table 7:** ImageJ porosity analysis of the as-built samples.

Sample	Average Porosity (%)	Average Pores/mm^2^	Average Feret Size (µm)
1	9.18	38.27	39.53
2	0.64	5.69	61.52
3	0.31	2.36	190.37
4	2.71	40.68	30.63
5	0.45	6.79	70.72
6	0.14	2.80	108.30
7	5.15	44.94	32.90
8	0.09	5.64	46.80
9	0.28	18.37	14.06
10	3.50	47.99	29.99
11	0.07	13.76	8.82
12	2.70	24.56	39.37
13	3.49	28.74	38.74
14	0.06	3.12	15.01
15	0.39	4.11	35.21
16	2.83	30.30	43.42
17	0.06	6.73	11.32
18	0.46	11.46	30.38
19	1.51	29.11	32.84
20	0.50	12.56	39.55
21	6.41	63.72	44.59
22	1.38	31.03	27.33
23	0.63	32.54	20.41
24	5.98	63.86	38.42
25	1.21	28.70	25.06
26	0.10	2.24	78.23
27	1.85	36.18	36.99

## Data Availability

The original contributions presented in the study are included in the article, further inquiries can be directed to the corresponding author.

## Appendix A

**Table A1:** ImageJ porosity analysis for the XZ and XY planes of the as-built samples.

Sample	Porosity (%)	Pores/mm^2^	Average Feret Size (µm)
1-XZ	9.93	39.26	37.23
1-XY	8.43	37.28	41.83
**1-Average**	**9.18**	**38.27**	**39.53**
2-XZ	0.72	4.78	34.25
2-XY	0.57	6.60	88.79
**2-Average**	**0.64**	**5.69**	**61.52**
3-XZ	0.22	2.64	63.54
3-XY	0.40	2.07	317.20
**3-Average**	**0.31**	**2.36**	**190.37**
4-XZ	3.22	33.17	31.99
4-XY	2.21	48.20	29.26
**4-Average**	**2.71**	**40.68**	**30.63**
5-XZ	0.54	2.91	92.30
5-XY	0.35	10.66	49.14
**5-Average**	**0.45**	**6.79**	**70.72**
6-XZ	0.18	2.29	83.92
6-XY	0.10	3.31	132.67
**6-Average**	**0.14**	**2.80**	**108.30**
7-XZ	7.78	42.51	35.32
7-XY	2.51	47.37	30.48
**7-Average**	**5.15**	**44.94**	**32.90**
8-XZ	0.07	6.48	11.87
8-XY	0.11	4.80	81.73
**8-Average**	**0.09**	**5.64**	**46.80**
9-XZ	0.45	7.75	20.15
9-XY	0.12	28.99	7.98
**9-Average**	**0.28**	**18.37**	**14.06**
10-XZ	4.59	52.47	25.93
10-XY	2.41	43.52	34.05
**10-Average**	**3.50**	**47.99**	**29.99**
11-XZ	0.08	9.53	10.24
11-XY	0.06	18.00	7.39
**11-Average**	**0.07**	**13.76**	**8.82**
12-XZ	2.77	15.18	48.80
12-XY	2.64	33.94	29.95
**12-Average**	**2.70**	**24.56**	**39.37**
13-XZ	4.84	23.63	36.87
13-XY	2.13	33.85	40.61
**13-Average**	**3.49**	**28.74**	**38.74**
14-XZ	0.04	2.76	13.86
14-XY	0.09	3.49	16.17
**14-Average**	**0.06**	**3.12**	**15.01**
15-XZ	0.68	5.43	30.25
15-XY	0.10	2.79	40.17
**15-Average**	**0.39**	**4.11**	**35.21**
16-XZ	3.21	28.36	39.94
16-XY	2.46	32.24	46.91
**16-Average**	**2.83**	**30.30**	**43.42**
17-XZ	0.08	7.79	12.96
17-XY	0.05	5.66	9.69
**17-Average**	**0.06**	**6.73**	**11.32**
18-XZ	0.35	9.51	21.61
18-XY	0.56	13.42	39.15
**18-Average**	**0.46**	**11.46**	**30.38**
19-XZ	1.55	35.86	23.25
19-XY	1.48	22.36	42.43
**19-Average**	**1.51**	**29.11**	**32.84**
20-XZ	0.54	8.36	56.31
20-XY	0.46	16.75	22.78
**20-Average**	**0.50**	**12.56**	**39.55**
21-XZ	6.83	54.49	45.66
21-XY	5.99	72.95	43.52
**21-Average**	**6.41**	**63.72**	**44.59**
22-XZ	1.37	34.71	28.54
22-XY	1.40	27.35	26.13
**22-Average**	**1.38**	**31.03**	**27.33**
23-XZ	0.71	32.37	21.38
23-XY	0.54	32.72	19.45
**23-Average**	**0.63**	**32.54**	**20.41**
24-XZ	5.58	62.21	38.60
24-XY	6.38	65.51	38.23
**24-Average**	**5.98**	**63.86**	**38.42**
25-XZ	1.38	36.03	23.94
25-XY	1.04	21.37	26.18
**25-Average**	**1.21**	**28.70**	**25.06**
26-XZ	0.06	2.84	15.05
26-XY	0.14	1.64	141.41
**26-Average**	**0.10**	**2.24**	**78.23**
27-XZ	2.22	23.71	49.40
27-XY	1.48	48.66	24.59
**27-Average**	**1.85**	**36.18**	**36.99**
